# A New Method for Surgical Treatment of Subcondylar Fractures: A Case Report

**Published:** 2017-09

**Authors:** Gholamreza Shirani, Mahnaz Arshad, Kamran Rasouli, Touraj Vaezi

**Affiliations:** 1 Assistant Professor, Department of Oral and Maxillofacial Surgery, School of Dentistry, Tehran University of Medical Sciences, Tehran, Iran; 2 Assistant Professor, Department of Prosthodontics, School of Dentistry, International Campus, Tehran University of Medical Sciences, Tehran, Iran; 3 Dentistry Student, School of Dentistry, International Campus, Tehran University of Medical Sciences, Tehran, Iran

**Keywords:** Fracture Fixation, Mandibular Condyle, Open Fracture Reduction, Maxillomandibular Fixation

## Abstract

Subcondylar fractures are common in the maxillofacial region following direct trauma to the mandibular ramus. The literature is replete with articles written on the treatment of subcondylar fractures, encompassing a plethora of various surgical approaches; however, the best treatment procedure has remained controversial. Such fractures are either treated by open reduction with internal fixation or closed reduction with maxillomandibular fixation. In this article, we describe a new surgical method for treatment of subcondylar fractures.

## INTRODUCTION

Mandibular fractures rank third after nasal and zygomatic fractures. Mandibular condylar neck fractures and subcondylar fractures comprise about 19–29% and 62–70% of all mandibular fractures, respectively [1]. The etiologies of maxillofacial fractures are influenced by cultural and environmental factors and vary among different countries. Overall, road accidents, fights, falls and sports injuries are the main reasons of subcondylar fractures [2]. Proper treatment of the subcondylar fracture is essential for maintenance of speech, eating, swallowing and masticatory function. Immobilization, good blood supply and appropriate alignment of the fractured bone fragments are mandatory for primary and secondary bone healing. In other words, appropriate reduction and fixation are critical for achieving satisfactory postoperative results [3]. Open treatment of subcondylar fractures is difficult and is a controversial issue in maxillofacial surgery. Although many procedures are available for treatment of these fractures, none is optimal [4], as the treatment results vary and each of these methods has some advantages and disadvantages. Different treatment options for mandibular fractures have been described in adults, such as:

1) Closed reduction with maxillomandibular fixation, 2) Open reduction with internal fixation, and 3) Endoscopic-assisted reduction with internal fixation [5]. Selection of a treatment modality for subcondylar fracture is controversial and depends on the displacement severity, fracture area and factors such as the patient’s age and coexistence of other fractures [6]. Intermaxillary fixation is performed by the use of the arch bar and wire for 2 to 4 weeks in the closed reduction approach. After stabilization of the fracture site, intermaxillary fixation is removed and normal occlusion is maintained by the use of rubber bands and a soft diet for 2 weeks. Functional therapy is performed simultaneously to restore the previous state of mandibular movement.

The duration of initial intermaxillary fixation is different case by case. Closed reduction is considered a safe treatment since it does not damage the nerves and blood vessels and causes no postoperative complications or residual scars [6]. Prolonged intermaxillary fixation may injure periodontal tissues, disturb oral hygiene maintenance and cause speech and respiratory complications. Inaccurate reduction of bone fragments may lead to growth disorders, overgrowth of the fractured bone, mandibular deviation and facial and temporomandibular joint (TMJ) asymmetry in children [6]. Open reduction is performed through various surgical methods depending on the fracture site and number of bone fragments. The methods include pre-auricular, post-auricular, submandibular, combined, and retromandibular approaches [5–7].

Incision-making in the open reduction method, to access the fractured bone, is considered invasive as it may lead to the injury of nerves and vessels or other complications during surgery. The advantages of the open reduction method include ideal reduction of the fractured bone to the correct anatomical position, provision of a direct access to the fracture site and improved bone fusion by use of plates and screws. Favorable postoperative TMJ function, optimal bone healing and faster rehabilitation of mandibular function may be observed.

Complications can be avoided by a short duration of intermaxillary fixation [6–8]. However, the open reduction may cause postoperative complications such as infection and Bell’s palsy, and it may also leave a permanent scar. In the present article, we describe a new surgical method for treatment of subcondylar fractures.

## CASE REPORT

**Fig. 1:**
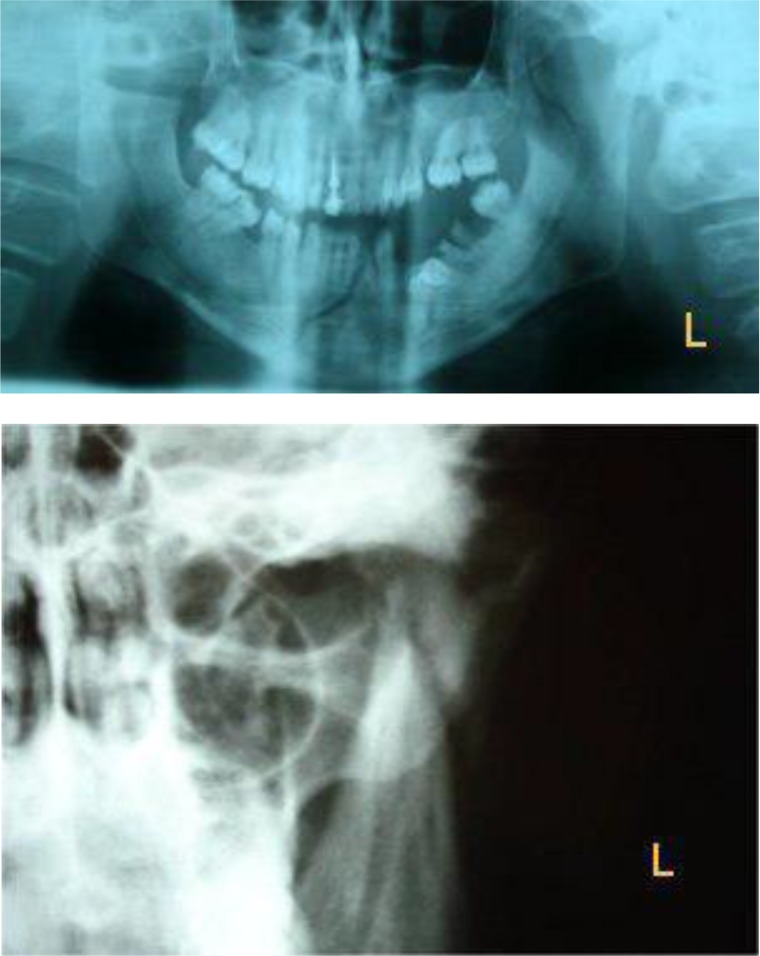
Subcondylar fracture. (a) Panoramic view. (b) Posteroanterior (PA) mandibular view

A 30-year-old man with a subcondylar fracture due to a car accident was referred to the oral and maxillofacial surgery department of Tehran University of Medical Sciences. The panoramic radiograph supplemented with a posteroanterior (PA) mandibular view revealed a fracture line located under the neck of the left mandibular condylar process. The fractured bone had been laterally displacement due to the contraction of the temporalis muscle (Fig. 1).

**Fig. 2:**
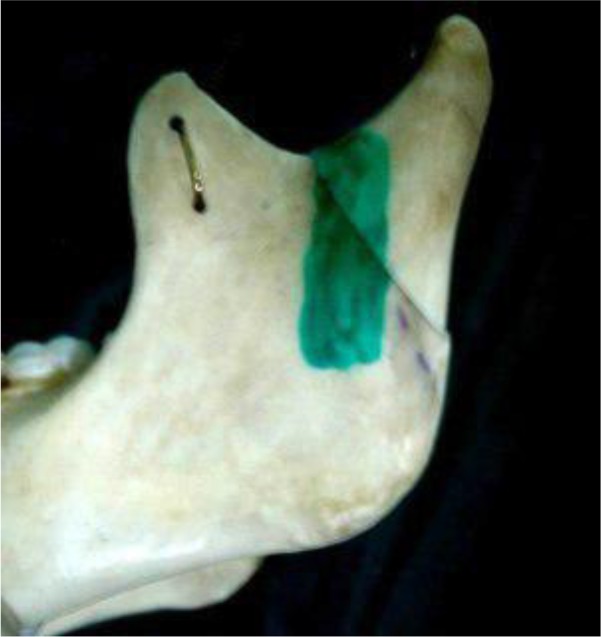
Osteoreduction line in the Shirani method

**Fig. 3:**
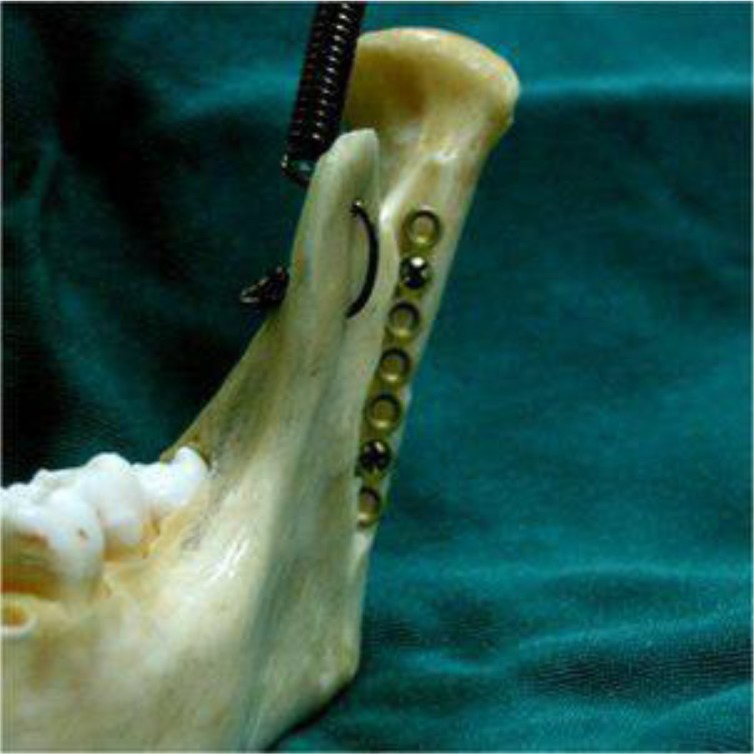
Bone fixation with plate and screws in the Shirani method

**Fig. 4:**
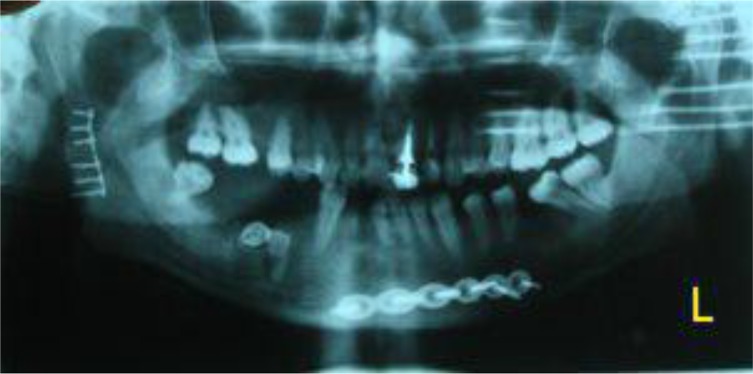
Postoperative panoramic view

In the operating room, an intraoral incision was made in the soft tissue similar to that performed in sagittal osteotomy (approximately 1cm above the dental occlusal surface, from the anterior aspect of the ramus to the mesial aspect of the second molar). After soft tissue dissection and accessing the fractured bone, a vertical step was created on the ramus body extending from the mandibular notch to the superior aspect of the angle of the mandible using an end cutting fissure bur (Fig. 2). After repositioning the fractured bone, a plate was secured in the rectangular-shaped step and was fixed by two screws (Fig. 3). Maxillomandibular rigid fixation was performed using the arch bar and wire for one week, followed by a two-week non-rigid fixation with elastics for occlusal adjustment. After 48 hours, panoramic and PA mandibular views were used to assess the results of the surgery. To ensure that the procedure has been properly done, mouth opening, occlusion, and TMJ function were assessed (Fig. 4). One-year follow-up showed no complications or canting, and the patient was satisfied with the results.

## DISCUSSION

Condylar and subcondylar fractures are among the most frequent fractures caused by car accidents. Untreated fractures may cause facial growth disturbances, TMJ disorders (e.g. ankylosis and dysfunction) and aesthetic problems. Different factors may interfere with the treatment of subcondylar fractures such as the patient’s age and concurrent unilateral or bilateral fractures in mandibular or maxillary bones [9].

The advantages and disadvantages of various approaches used for management of subcondylar fractures have been previously evaluated [5]. Closed reduction with intermaxillary fixation is a non-invasive approach widely used to treat condylar and subcondylar fractures. It poses a minor danger to the facial nerve and leaves no scars. Some complications may arise in closed reduction such as inappropriate vertical high that may lead to malocclusion, improper anatomical reduction, and weight loss because of intermaxillary fixation. Another issue is the duration of maxillomandibular fixation; it may vary from 2 to 6 weeks (a two-week rigid fixation followed by elastic fixation in case of malocclusion). Surgeons prefer short fixation periods to avoid problems such as TMJ ankylosis. Trauma to the TMJ capsule can also induce TMJ ankylosis. This complication of the closed reduction approach compelled surgeons to seek new methods to treat fractures [5]. In the Shirani method, the surgeon makes an intraoral incision with a low risk of facial nerve damage and without any residual scars. Intraoral access to the fracture site by the Shirani method is more difficult compared to the extraoral approach. The advantages of this new method over the closed reduction include direct access to the fracture site, improved alignment of the fractured bones, satisfactory mandibular and TMJ function, suitable postoperative occlusion and short-term maxillomandibular fixation.

## CONCLUSION

The intraoral incision differentiates closed reduction with the Shirani method from the open reduction approach. The advantages of the Shirani method include minimum risk of damage to the facial nerve and parotid gland, no residual scars, anatomic fixation and reduced period of maxillomandibular fixation with elastics.
